# Supplementary material for: Pyruvate Dehydrogenase Kinase 1 inhibition mediated oxidative phosphorylation enhancement in cartilage promotes osteoarthritis progression
Source: BMC Musculoskelet Disord. 2023 Jul 20;24:597. doi: 10.1186/s12891-023-06585-6 (PMC10357736; doi:10.1186/s12891-023-06585-6)
Supplement: Supplementary file 1 — Additional file 1. [file 12891_2023_6585_MOESM1_ESM.zip › KEGG.Gsea.1653622432464/KEGG_GLYCOSAMINOGLYCAN_BIOSYNTHESIS_CHONDROITIN_SULFATE.html]

Details for gene set KEGG\_GLYCOSAMINOGLYCAN\_BIOSYNTHESIS\_CHONDROITIN\_SULFATE[GSEA]

|  || Dataset | 55457\_collapsed\_to\_symbols.cls.cls #OA\_versus\_normal.cls.cls #OA\_versus\_normal\_repos |
| Phenotype | cls.cls#OA\_versus\_normal\_repos |
| Upregulated in class | OA |
| GeneSet | KEGG\_GLYCOSAMINOGLYCAN\_BIOSYNTHESIS\_CHONDROITIN\_SULFATE |
| Enrichment Score (ES) | 0.34104115 |
| Normalized Enrichment Score (NES) | 1.0590708 |
| Nominal p-value | 0.36829838 |
| FDR q-value | 0.5028336 |
| FWER p-Value | 1.0 |
Table: GSEA Results Summary

  

Fig 1: Enrichment plot: KEGG\_GLYCOSAMINOGLYCAN\_BIOSYNTHESIS\_CHONDROITIN\_SULFATE      
 Profile of the Running ES Score & Positions of GeneSet Members on the Rank Ordered List

  

| SYMBOL | TITLE | RANK IN GENE LIST | RANK METRIC SCORE | RUNNING ES | CORE ENRICHMENT || 1 | CHST12 | carbohydrate sulfotransferase 12 [Source:HGNC Symbol;Acc:HGNC:17423] | 985 | 0.521 | 0.0487 | Yes |
| 2 | B4GALT7 | "beta-1,4-galactosyltransferase 7 [Source:HGNC Symbol;Acc:HGNC:930]" | 1411 | 0.442 | 0.1218 | Yes |
| 3 | XYLT2 | xylosyltransferase 2 [Source:HGNC Symbol;Acc:HGNC:15517] | 1938 | 0.368 | 0.1694 | Yes |
| 4 | CHPF2 | chondroitin polymerizing factor 2 [Source:HGNC Symbol;Acc:HGNC:29270] | 2281 | 0.329 | 0.2218 | Yes |
| 5 | CSGALNACT1 | chondroitin sulfate N-acetylgalactosaminyltransferase 1 [Source:HGNC Symbol;Acc:HGNC:24290] | 2507 | 0.302 | 0.2770 | Yes |
| 6 | B3GAT3 | "beta-1,3-glucuronyltransferase 3 [Source:HGNC Symbol;Acc:HGNC:923]" | 2987 | 0.251 | 0.3001 | Yes |
| 7 | UST | uronyl 2-sulfotransferase [Source:HGNC Symbol;Acc:HGNC:17223] | 3180 | 0.233 | 0.3410 | Yes |
| 8 | XYLT1 | xylosyltransferase 1 [Source:HGNC Symbol;Acc:HGNC:15516] | 3916 | 0.163 | 0.3231 | No |
| 9 | CHST11 | carbohydrate sulfotransferase 11 [Source:HGNC Symbol;Acc:HGNC:17422] | 4664 | 0.099 | 0.2889 | No |
| 10 | B3GAT1 | "beta-1,3-glucuronyltransferase 1 [Source:HGNC Symbol;Acc:HGNC:921]" | 4715 | 0.095 | 0.3078 | No |
| 11 | CHST3 | carbohydrate sulfotransferase 3 [Source:HGNC Symbol;Acc:HGNC:1971] | 5169 | 0.058 | 0.2866 | No |
| 12 | CHST7 | carbohydrate sulfotransferase 7 [Source:HGNC Symbol;Acc:HGNC:13817] | 6486 | -0.041 | 0.1944 | No |
| 13 | DSE | dermatan sulfate epimerase [Source:HGNC Symbol;Acc:HGNC:21144] | 7493 | -0.118 | 0.1446 | No |
| 14 | CHPF | chondroitin polymerizing factor [Source:HGNC Symbol;Acc:HGNC:24291] | 7960 | -0.152 | 0.1449 | No |
| 15 | CHST15 | carbohydrate sulfotransferase 15 [Source:HGNC Symbol;Acc:HGNC:18137] | 8220 | -0.173 | 0.1663 | No |
| 16 | CSGALNACT2 | chondroitin sulfate N-acetylgalactosaminyltransferase 2 [Source:HGNC Symbol;Acc:HGNC:24292] | 9773 | -0.298 | 0.1174 | No |
| 17 | CHSY1 | chondroitin sulfate synthase 1 [Source:HGNC Symbol;Acc:HGNC:17198] | 11628 | -0.521 | 0.0987 | No |
Table: GSEA details [plain text format]

  

Fig 2: KEGG\_GLYCOSAMINOGLYCAN\_BIOSYNTHESIS\_CHONDROITIN\_SULFATE      
 Blue-Pink O' Gram in the Space of the Analyzed GeneSet

  

Fig 3: KEGG\_GLYCOSAMINOGLYCAN\_BIOSYNTHESIS\_CHONDROITIN\_SULFATE: Random ES distribution      
 Gene set null distribution of ES for **KEGG\_GLYCOSAMINOGLYCAN\_BIOSYNTHESIS\_CHONDROITIN\_SULFATE**
